# Development of Chromatographic Fingerprints of *Eurycoma longifolia* (Tongkat Ali) Roots Using Online Solid Phase Extraction-Liquid Chromatography (SPE-LC)

**DOI:** 10.3390/molecules21050583

**Published:** 2016-04-30

**Authors:** Nor Nasriah Zaini, Rozita Osman, Hafizan Juahir, Norashikin Saim

**Affiliations:** 1 Faculty of Applied Sciences, Universiti Teknologi MARA, Shah Alam 40450, Selangor, Malaysia; nasriah_zaini@yahoo.com (N.N.Z.); rozit471@salam.uitm.edu.my (R.O.); 2 East Coast Environmental Research Institute, Universiti Sultan Zainal Abidin, Kuala Terengganu 21300, Malaysia; hafizanjuahir@unisza.edu.my

**Keywords:** chromatographic fingerprint, eurycomanone, *Eurycoma longifolia*, online SPE-LC, cluster analysis, discriminant analysis, principal component analysis

## Abstract

*E. longifolia* is attracting interest due to its pharmacological properties and pro-vitality effects. In this study, an online SPE-LC approach using polystyrene divinyl benzene (PSDVB) and C18 columns was developed in obtaining chromatographic fingerprints of *E. longifolia*. *E. longifolia* root samples were extracted using pressurized liquid extraction (PLE) technique prior to online SPE-LC. The effects of mobile phase compositions and column switching time on the chromatographic fingerprint were optimized. Validation of the developed method was studied based on eurycomanone. Linearity was in the range of 5 to 50 µg∙mL^−1^ (r^2^ = 0.997) with 3.2% relative standard deviation of peak area. The developed method was used to analyze 14 *E. longifolia* root samples and 10 products (capsules). Selected chemometric techniques: cluster analysis (CA), discriminant analysis (DA), and principal component analysis (PCA) were applied to the fingerprint datasets of 37 selected peaks to evaluate the ability of the chromatographic fingerprint in classifying quality of *E. longifolia*. Three groups were obtained using CA. DA yielded 100% correlation coefficient with 19 discriminant compounds. Using PCA, *E. longifolia* root samples were clearly discriminated from the products. This study showed that the developed online SPE-LC method was able to provide comprehensive evaluation of *E. longifolia* samples for quality control purposes.

## 1. Introduction

Herbal formulations are gaining popularity worldwide for health promotion and adjuvant therapy. Many studies based on experimental evidence have reported the pharmacological properties of *Eurycoma longifolia*, including anti-malarial, anti-diabetic, anticancer, antiulcer and aphrodisiac effects [1,2,3,4,5,6,7,8]. Due to these effects, *E. longifolia* extracts are commonly used as an alternative medicine or dietary supplements making this species one of the most popular herbal plants. As the demand of this plant is increasing globally [1], a fast and reliable method for determining its quality is required.

Quality control is crucial in ensuring safety and efficacy of herbal formulations [7]. A comprehensive evaluation of the chemical profile, instead of only a single compound [9] is essential. A chromatographic fingerprint with specific profile or pattern based on detected compounds has been successfully applied in quality control of herbal formulation [9,10,11]. Chromatographic fingerprint technology has gained increasing attention and has been accepted by leading organizations such as the World Health Organization (WHO), Federal Drug Administration (FDA) and the British Herbal Medicine Association [12,13].

Due to the chemical complexity of herbal plants extract [14], an efficient analytical methodology for revealing chemical fingerprints is of great and current interest [3]. Common approaches involve several steps, including extraction (soaking [6], Soxhlet [4], PLE [15,16]), clean up using SPE [17,18] followed by gas chromatography (GC) [19] or high performance liquid chromatography (HPLC) [3,13]. In this study, online solid phase extraction-liquid chromatography (SPE-LC) method was developed in obtaining comprehensive fingerprints for *E. longifolia*. Using online SPE-LC, sample clean-up and HPLC analysis were achieved simultaneously by column switching strategy, designed to increase throughput and provide sufficient resolving power [20]. Online SPE-LC provides rapid analyses for complex mixtures.

The chromatographic performance of the online SPE-LC system, including accuracy, precision and repeatability was estimated by analyzing eurycomanone, the major compound in *E. longifolia*. The attributes of the chromatographic fingerprint obtained using the developed online SPE-LC in quality control of *E. longifolia* was evaluated by chemometric approaches using 37 selected peaks. Cluster analysis (CA) and principal component analysis (PCA) are the most common chemometric methods used in quality evaluation of herbal fingerprints [21,22].

## 2. Results and Discussion

### 2.1. Optimization of Online SPE-LC Conditions

**Figure 1:**
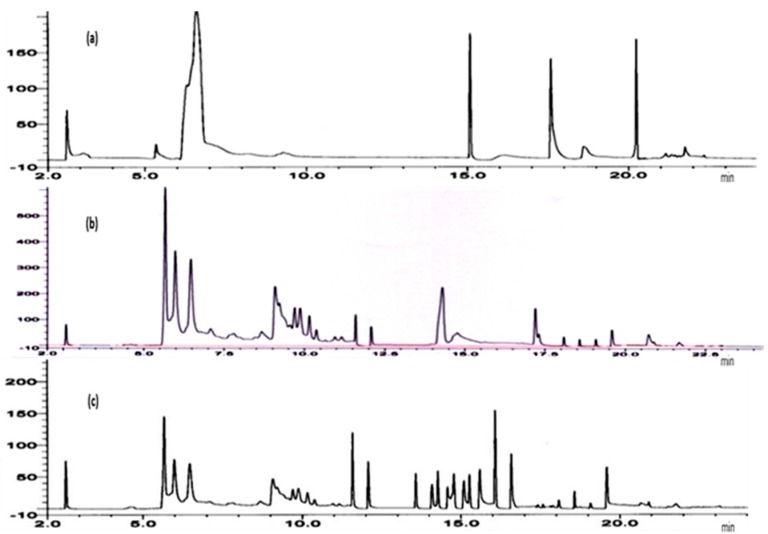
Chromatographic fingerprints of *E. longifolia* roots. (**a**) 0–6.0 min: 80% A:20% C; 6.0–16.0 min: 50% A:50% B; 16.0–25.0 min; 70% B:30% C; (**b**) 0–5.7 min: 70% A:30% C ; 5.7–8.5 min gradient elution to 30% A:70% C; 8.5–16.0 min: gradient elution 30% A:70% B to 60% A:40% B; 16.0–19.0 min: gradient elution 50% B:50% C to 10% B:90% C; 19.0–25.0 min; isocratic 10% B:90% C; (**c**) conditions same as (**b**) with column switching between 12–18 min.

Reverse phase-high performance liquid chromatography (RP-HPLC) is a predominant technique for the separation of eurycomanone in *E. longifolia* [4] after clean up using SPE C18 [17]. In this study, the chromatographic fingerprint of *E. longifolia* root extract obtained by the online SPE-LC technique was developed using PSDVB as the SPE column and C18 as the separating column. Methanesulfonic acid (MSA) (95%) and ultrapure water (5%) were used as eluting solvent for SPE. Various gradient elution compositions of mobile phase comprising ultrapure water (A), methanol (B) and acetonitrile (C) were studied. A decrease in the composition of ultrapure water with an increase of ACN (70% A and 30% C) improved the separation of the compounds (Figure 1a,b). The quality of the chromatographic fingerprint was further improved by optimizing the column switching time, defined as the time set for elution of analytes from the SPE column. Column switching time will affect the removal of interferences from the matrix [23]. Optimizing column switching time is important at optimum HPLC gradient elution program in producing better separation of the analytes [24]. An increase in the number of separated compounds was observed with column switching between 12–18 min (Figure 1c).

### 2.2. Method Validation of Online SPE-LC

**Figure 2:**
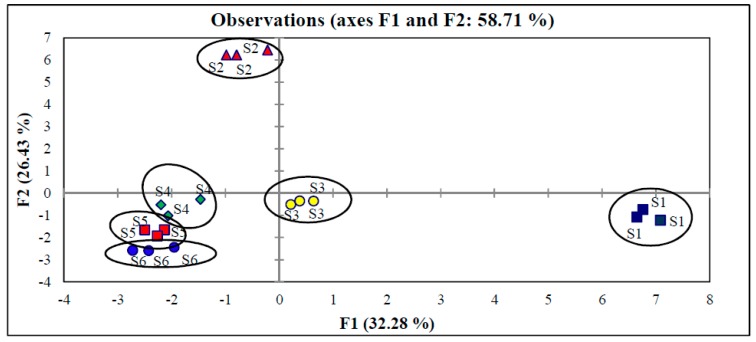
PCA plot on triplicate analysis of *E. longifolia* roots samples.

In order for an analytical method to be employed and regarded as reliable, method validation must be carried out. In this study, a standard solution of eurycomanone (50 µg∙mL^−1^) was used in the determination of accuracy and precision of the developed method. High recovery (90.6%) of eurycomanone was obtained and ten replicates of standard eurycomanone gave low percent RSD (3.7%). Analysis of six *E. longifolia* root extract showed good repeatability (2.5% RSD). Good reproducibility was supported by principal component analysis (PCA) of 37 selected compounds (based on peak areas) from the chromatographic fingerprint. Triplicate analysis of each *E. longifolia* root extract clustered close together while distinct separation between the six types of *E. longifolia* root samples were observed (Figure 2).

Linearity study was conducted using standard eurycomanone with concentration ranges from 5 to 50 µg∙mL^−1^. Linear relationship of the correlation coefficients (R^2^= 0.997) was obtained with linear regression equation y = 0.0476x + 1.5654. The limit of detection (LOD) and quantification (LOQ) were identified by injecting a series of reference standard of eurycomanone until the signal-to-noise (S/N) ratio was 3 and 10 for LOD and LOQ, respectively. LOD and LOQ of eurycomanone were 2.7 µg∙mL^−1^ and 9.1 µg∙mL^−1^, respectively.

### 2.3. Chromatographic Fingerprint of E. longifolia Root Samples

**Figure 3:**
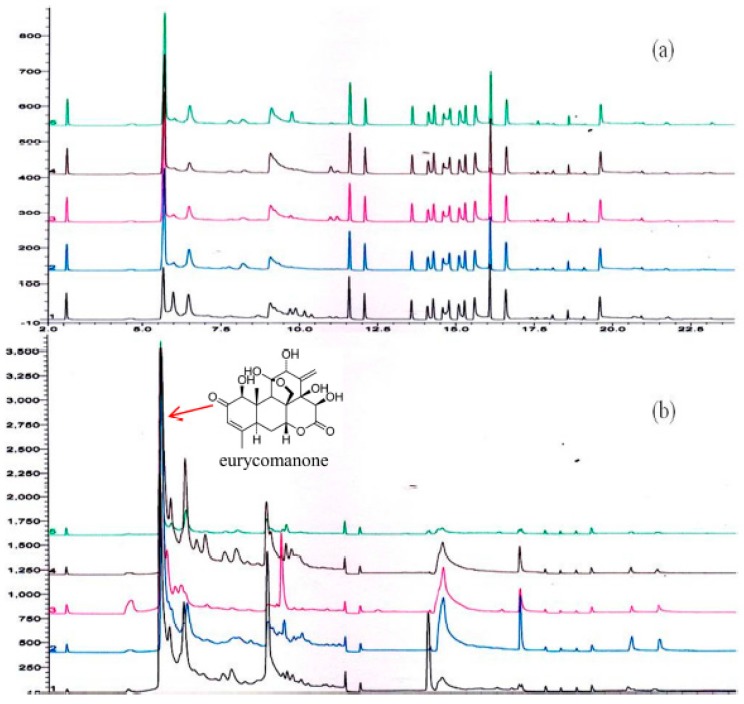
Chromatographic fingerprints of *E. longifolia* (**a**) roots (**b**) products.

Chromatographic fingerprints of five *E. longifolia* root samples (Figure 3a) and five of its products (Figure 3b) were obtained using the developed online SPE-LC. Major quassinoids in the root of *E. longifolia* are eurycomanone and eurycomanol [25]. Eurycomanone, identified as the most representative quassinoid in *E. longifolia* root extracts [26] was eluted at retention time of 5.6 min. The chromatographic fingerprints obtained visibly showed similarities and differences among the *E. longifolia* roots and products.

### 2.4. Chemometric Methods

Fourteen *E. longifolia* roots samples (R1 to R14) from four states in Malaysia and 10 products (P1 to P10) from different manufacturers were chosen as representative samples. All samples were analyzed using an optimized sample preparation method under optimized online SPE-LC conditions. The obtained peak areas in the fingerprints were used as explanatory variables. Peak areas of 37 peaks selected based on reproducible retention times (RSD < 2.5%) and peak areas (RSD 5.0%) were utilized to establish pattern recognition in fingerprint analysis [21,27,28,29].

#### 2.4.1. Cluster Analysis (CA)

**Figure 4:**
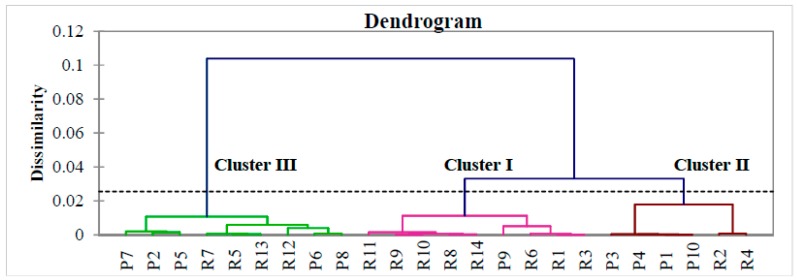
Dendrogram of *E. longifolia* roots and products.

CA was applied to the pattern recognition of 14 batches of *E. longifolia* roots and 10 of its products in order to classify 37 variables using between-groups linkages as amalgamation rule (similarities) and Euclidean distance metric (dissimilarities). The dendrogram (Figure 4) visibly showed that all samples were divided into three clusters. From the eight root samples in cluster 1, four (R8 to R11) were obtained from the same state. In addition, two root samples from the same state were clustered together in cluster 2. The results suggested that the obtained chromatographic fingerprints may be used as source determination. The clustering of products in the same group with the roots may suggest that they were from the same source. As the products are formulated using *E. longifolia* extract, the extraction solvent used may affect the fingerprint composition [3] and the cluster analysis.

#### 2.4.2. Discriminant Analysis (DA)

**Figure 5:**
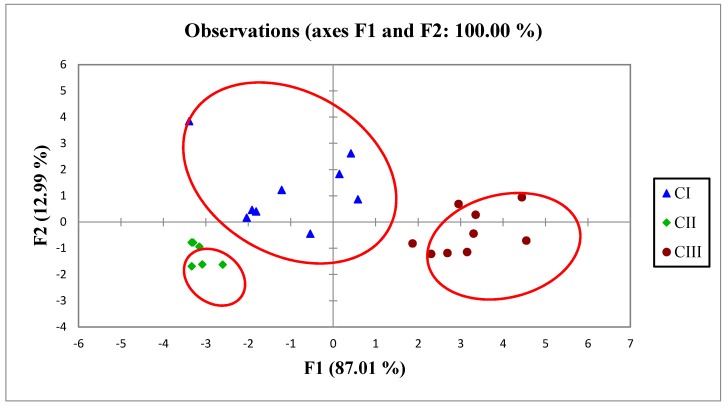
Discriminant analysis of the sampling groups.

The three clusters obtained from CA were considered as dependent variables (CI, CII, CIII) to DA. DA showed that each group differed from the others in terms of different compositions as the no confusion matrix occurs on the data set [30]. The results from standard, stepwise backward and stepwise forward modes gave 100% correct classification based on the confusion matrix of the estimation sample (Figure 5).

Stepwise discriminant analysis was performed as an explanatory analysis to determine the most significant variables among the parameters. In stepwise forward mode, variables were included one by one, beginning with the most significant until no significant changes were obtained, while in stepwise backward mode, variables were removed one by one beginning with the least significant until no significant changes were obtained.

Stepwise backward mode yielded 100% correctly with 19 discriminant variables (peaks) whereas stepwise forward mode rendered 100% with only one discriminant variable, with little difference in matching for each sample compared with the stepwise backward mode. Therefore, DA results suggested all 19 discriminant variables as significant in discriminating the quality of *E. longifolia*.

#### 2.4.3. Principal Component Analysis (PCA)

**Figure 6:**
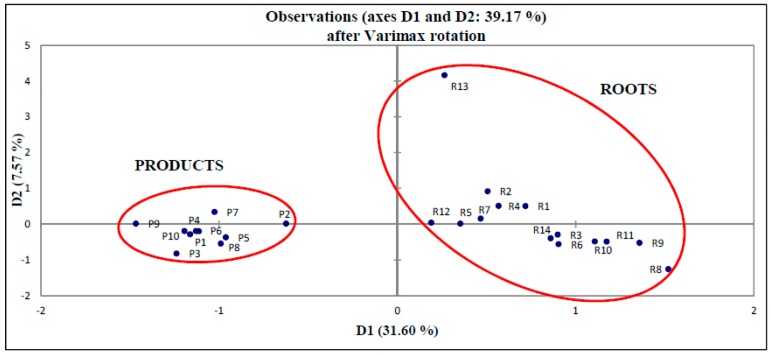
PCA plot of *E. longifolia.*

PCA is a powerful tool to reduce the dimensions of multivariate data set. In the present study, the RPA of 37 selected peaks were treated as variables. The first two PCs (PC1 and PC2) were selected to provide the highest variation of data objects (31.60% and 7.57% of the variation) for convenient visualization and differentiation. Figure 6 showed that the *E. longifolia* roots and products were distinctively separated. The result suggested that roots and products have their own unique chemical compositions.

## 3. Experimental Section

### 3.1. Chemicals, Reagents and Materials

Eurycomanone reference compound was purchased from Chromadex (Irvine, CA, USA). Acetonitrile (ACN) and methanol (MeOH) of HPLC grade were purchased from Merck (Darmstadt, Germany). Methanesulfonic acid (MSA) was purchased from Merck Schuchardt (Hohenbrunn, Germany). Roots of *E. longifolia* from various sources were obtained from local suppliers. Products (capsules) containing of *E. longifolia* were purchased from various manufacturers (registered with National Pharmaceutical Control Bureau, Selangor, Malaysia).

### 3.2. Sample Preparation

Pressurized Liquid Extraction (PLE) was performed on a Dionex ASE 350 accelerated solvent extractor (Thermo Scientific Ltd. Camberly, Surrey, UK). Dried root of *E. longifolia* was weighed (2.0 g) and mixed with an equal amount of diatomaceous earth, then transferred to a 34 mL PLE stainless steel extraction cell. Extraction was carried out at 100°C and 1500 psi for 30 min [31]. *E. longifolia* product (1.1 g) was dissolved in 15 mL of hot water (~80°C) and filtered using filter paper prior to online SPE-LC analysis.

### 3.3. Online Solid Phase Extraction Liquid Chromatography (SPE-LC)

Online SPE-LC analysis was performed on a Dionex Ultimate 3000 Liquid Chromatography system equipped with degasser, quaternary delivery system, an auto-sampler, column oven and a diode array detector (DAD). Online SPE-LC was done using polystyrene divinyl benzene (PSDVB) column (5 µm, 4.6 mm × 50 mm), while all the chromatographic separation was carried out on a C18 column (5 µm, 4.6 mm × 250 mm). The column temperature was maintained at 37 °C in an oven and injection volume was 100 µL. Data acquisition was performed by Chromeleon software (Version 6.8, Dionex, Sunnyvale, CA, USA).

The method involved four major steps: sample loading, clean up, elution of extract and LC separation achieved using two pumps (left and right) operated simultaneously. The left pump controlled the first three steps while the right pump was applied for separation of analytes. Solvents delivered by left pump were 95% MSA and 5% ultrapure water, while those of the right pump were ultrapure water (A), methanol (B) and acetonitrile (C). Both pumps were operated simultaneously.

### 3.4. Data Analysis

The chemometric techniques of cluster analysis (CA), principal component analysis (PCA) and discriminant analysis (DA) were performed using XLSTAT Software (XLSTAT, 2015, Addinsoft, New York, NY, USA) for statistical analysis. For this purpose, 37 peaks were selected and peak areas were used as variables.

#### 3.4.1. Principal Component Analysis

Principal component analysis (PCA) is one of the most popular methods used for data analysis. PCA compresses the size of data by simplifying, extracting and keeping only important information. PCA creates linear combinations of the original variables called the principal components, which describes the systematic patterns of variation between the samples. The PCs are orthogonal and the number of PCs is less than or equal to the number of original variables. The first principal component has the largest possible variance.

#### 3.4.2. Cluster Analysis

Cluster analysis (CA) is an unsupervised pattern recognition technique that can be used instead of PCA or in combination of PCA. The cluster analysis using Ward’s linkage was chosen in this study as the algorithm showed the best results for chromatographic fingerprint data. CA helped to find hidden similarities or dissimilarities between samples.

#### 3.4.3. Discriminant Analysis (DA)

DA is a supervised pattern recognition technique which is applied to construct a classifier model from a data matrix and known class information. For an acceptable model, a high percentage of correct classification should be obtained.

## 4. Conclusions

A fast, reliable and comprehensive chromatographic fingerprint of *E. longifolia* root was obtained using an online SPE-LC method after extraction using PLE. Optimization of the mobile phase compositions and column switching time produced chromatographic fingerprint with good precision, accuracy and reproducibility. *E. longifolia* root samples and products from various sources were analyzed using the developed online SPE-LC. Chemometric analysis was applied on *E. longifolia* for uncovering the relationship between variables. CA helped to uncover the similarities among the roots and products forming three clusters. DA confirmed the results of CA and showed that 100% correct assignation with 19 discriminant features to discriminate the *E. longifolia* roots and products. The results of PCA differentiated between root samples and products. This study suggested that chromatographic fingerprint of *E. longifolia* obtained using online SPE-LC was able to provide a comprehensive chemical profile which would result in their different efficacies, thus could be a promising approach for quality control application. Further analyses for the identification of marker compounds of *E. longifolia* are being conducted to provide a fast, reliable and cost effective method suitable for authentication of *E. longifolia* products.

## Abbreviations

The following abbreviations are used in this manuscript:

SPE-LC: Solid phase extraction-liquid extraction
PLE: Pressurised liquid extraction
PSDVB: Polystyrene divinyl benzene
CA: Cluster analysis
PCA: Principal component analysis
DA: Discriminant analysis
WHO: World Health Organisation
FDA: Federal Drug Administration
GC: Gas chromatography
MSA: Methanesulfonic acid
ACN: Acetonitrile
RP-HPLC: Reverse phase-high performance liquid chromatography
LOD: Limit of detection
LOQ: Limit of quantification
S/N: Signal to noise
RSD: Relative standard deviation
RPA: Relative peak area
